# Epithelioid cell granuloma with caseating necrosis possibly caused by periapical periodontitis: a case report

**DOI:** 10.1186/s13256-018-1891-9

**Published:** 2018-12-11

**Authors:** Risa Shimizu, Kae Tanaka, Yu Oikawa, Hirofumi Tomioka, Kou Kayamori, Tohru Ikeda, Takatomo Yoshioka, Arata Ebihara, Hiroyuki Harada

**Affiliations:** 1 Department of Oral and Maxillofacial Surgery, Graduate School of Medical and Dental Sciences, Tokyo Medical and Dental University, 1-5-45 Yushima, Bunkyo-ku, Tokyo, 113-8549 Japan; 2 Section of Diagnostic Oral Pathology, Graduate School of Medical and Dental Sciences, Tokyo Medical and Dental University, 1-5-45 Yushima, Bunkyo-ku, Tokyo, 113-8549 Japan; 3 Yoshioka Dental Office, 2-3-13 Kanda-Surugadai, Chiyoda-ku, Tokyo, 101-0062 Japan; 4 Pulp Biology and Endodontics, Graduate School of Medical and Dental Sciences, Tokyo Medical and Dental University, 1-5-45 Yushima, Bunkyo-ku, Tokyo, 113-8549 Japan

**Keywords:** Epithelioid cell granuloma, Caseating necrosis, Periapical periodontitis

## Abstract

**Background:**

Epithelioid cell granuloma with caseating necrosis is a typical pathological finding in tuberculosis. While specific inflammation, including that related to tuberculosis, can induce caseating granuloma formation, there have been very few reports on the induction of caseating granuloma by non-specific inflammation. Chronic periapical periodontitis is usually related to bacterial biofilm formation as well as fungal or viral infection in the periapical lesion. However, it is difficult to eliminate these extraradicular pathogenic microbes by normal endodontic therapy alone, and more invasive surgical removal is almost always required.

**Case presentation:**

Here we describe the case of a 30-year-old Japanese woman who had suffered from dull pain related to periapical periodontitis for approximately 10 years. Although the causal tooth had been previously extracted at the Department of Oral Surgery of another hospital in 2015, inflammation of the surrounding tissue had not abated. She was referred to our hospital in May 2016 and underwent surgical debridement via an intra/extraoral approach under general anesthesia. A caseating granuloma accompanied by a small amount of fungi was histopathologically confirmed in the excised specimen. Her inflammation has not been exacerbated since the operation.

**Conclusions:**

This is the first report in which non-specific inflammation is shown to induce caseating granuloma arising in the jaw. Our report also highlights the importance of sufficient root canal treatment during the first stage of the procedure.

## Background

Epithelioid cell granuloma accompanied by Langhans multinucleated giant cells and enclosing caseating necrosis is a typical pathological finding in tuberculosis. In the head and neck area, tuberculosis commonly develops in the oral mucosa, especially the gingiva, and in the lymph nodes. Tubercles are rarely seen in the jaw, as tuberculous osteomyelitis occurs in < 2% of patients with skeletal tuberculosis [1]. However, epithelioid cell granuloma with caseating necrosis may also develop at sites of intense non-specific inflammation, albeit rarely. Furthermore, there has been no report of non-specific inflammation causing an epithelioid cell granuloma with caseating necrosis in the jaw, although involvement of the long bones has been reported.

Among the causes of persistent periapical periodontitis is endodontic failure due to defective instruments, perforations, overfilling, underfilling, ledges, and root canal therapies with iatrogenically altered root canal morphology [2]. In the affected teeth, bacterial biofilm formation as well as fungal and viral infections of the periapical lesion are likely [3–7]. These infections are difficult to eradicate by routine endodontic treatment, and surgical curettage, in some cases including tooth extraction, may be the only possible cure.

Here we describe a case of long-term periapical periodontitis in which overextended gutta-percha possibly induced the formation of an epithelioid cell granuloma with caseating necrosis.

## Case presentation

**Fig. 1:**
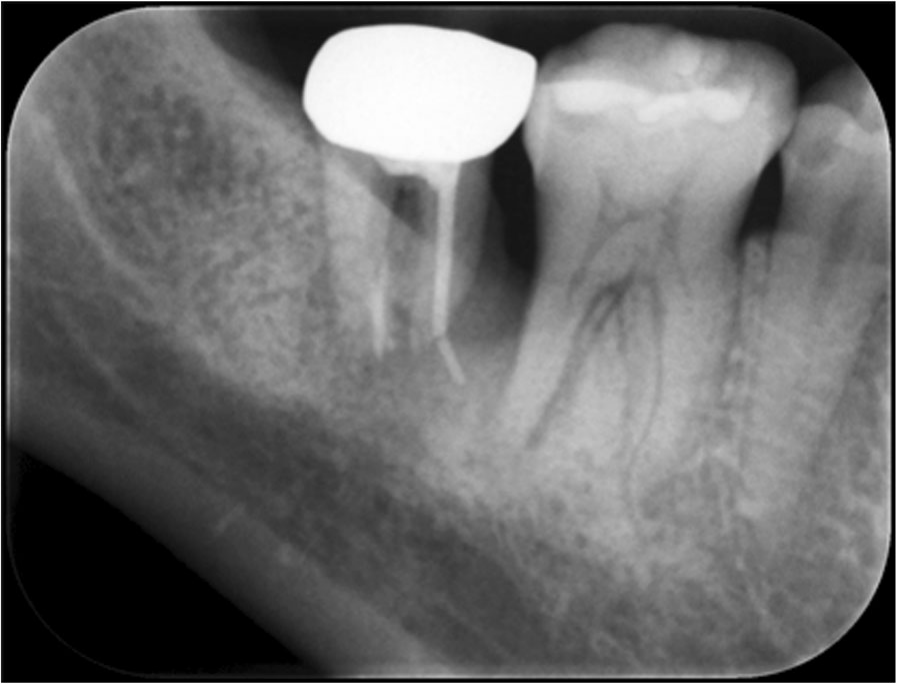
Periapical radiograph of tooth #47, showing insufficient root canal filling of the distal root, the extruded foreign body outside the apex of the mesial root, and radiolucency around both roots

**Fig. 2:**
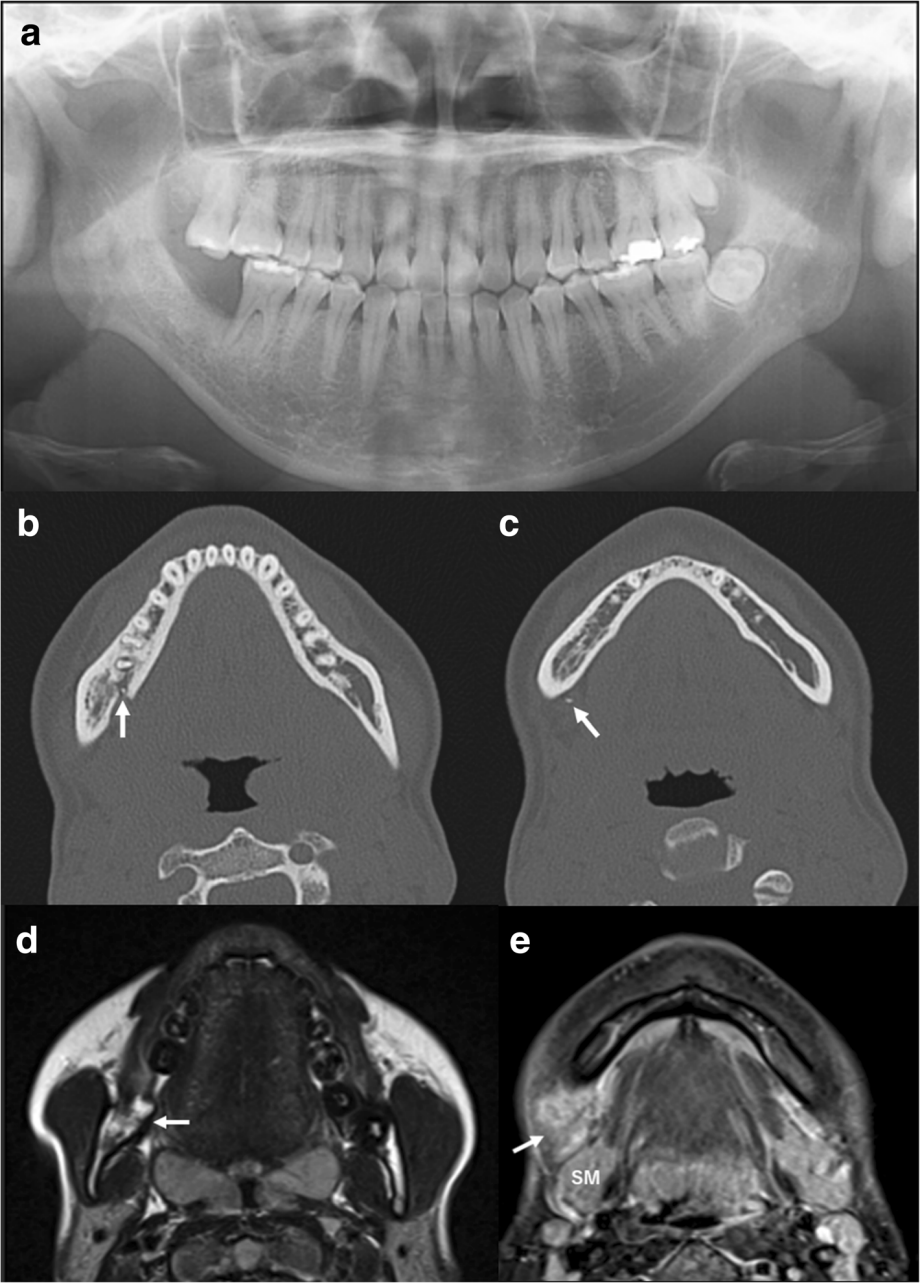
**a** Seven months after extraction of tooth #47, a panoramic radiograph showed new bone formation at the equivalent site of teeth #48 and #47 and a radiolucent region around the distal root of tooth #46. **b, c** Computed tomography showed continuous absorption from the alveolar bone of the distal root of tooth #46 to the lingual cortical bone at the equivalent of tooth #47 and some granulated hard tissues existing inside and outside the mandible (*arrow*). **d** Magnetic resonance imaging showed high intensity of the right lower molar region on an enhanced T2-weighted image (*arrow*). **e** The soft tissue located between interior to the mandibular angle and anterior to the submandibular gland was enhanced heterogeneously by gadolinium on magnetic resonance imaging (*arrow*). *SM* submandibular gland

A 19-year-old Japanese woman underwent root canal treatment on tooth #47 by a general practitioner in 2005, but dull pain persisted after. Tooth #48, which was horizontally embedded, was extracted in August 2010, but the pain continued. In August 2015, she presented at the Department of Oral Surgery of another hospital with a purulent discharge from the pocket of tooth #47. Intraoral radiography showed insufficient root canal filling in the distal root and a foreign body, suspected to be extruded gutta-percha, outside the apex of the mesial root of tooth #47, accompanied by bone absorption around both roots (Fig. 1). Two months later, she underwent extraction of tooth #47, removal of the foreign body, and curettage of the periapical lesion, resulting in the disappearance of the pain. However, she noted expansion of the right posterior mandibular bone in March 2016 and was thus referred in May 2016 to the Department of Oral and Maxillofacial Surgery, Tokyo Medical and Dental University, Japan. Her health and nutritional status were good in spite of a low body mass index (17.0). Both a blood test and a chest X-ray showed normal findings. She had neither lymphadenopathy nor paralysis of the mental/lingual nerve. The right inferior border of the mandibular bone slightly bulged. The socket of tooth #47 was epithelialized, and tooth #46 was vital, with a pocket depth of 3 mm. There was no sinus tract. Panoramic radiography revealed new bone formation at the equivalent sites of teeth #48 and #47 and a radiolucent region around the distal root of tooth #46 (Fig. 2a). Computed tomography showed continuous absorption from the alveolar bone of the distal root of tooth #46 to the lingual cortical bone at the equivalent of tooth #47 and six granulated hard tissues (Fig. 2b, c). On magnetic resonance imaging, the right lower molar region was of low intensity on an enhanced T1-weighted image and high intensity on an enhanced T2-weighted image (Fig. 2d). This same region was enhanced heterogeneously by gadolinium, as was the soft tissue located between interior to the mandibular angle and anterior to the submandibular gland (Fig. 2e). She was diagnosed clinically as having osteomyelitis of the right mandible.

**Fig. 3:**
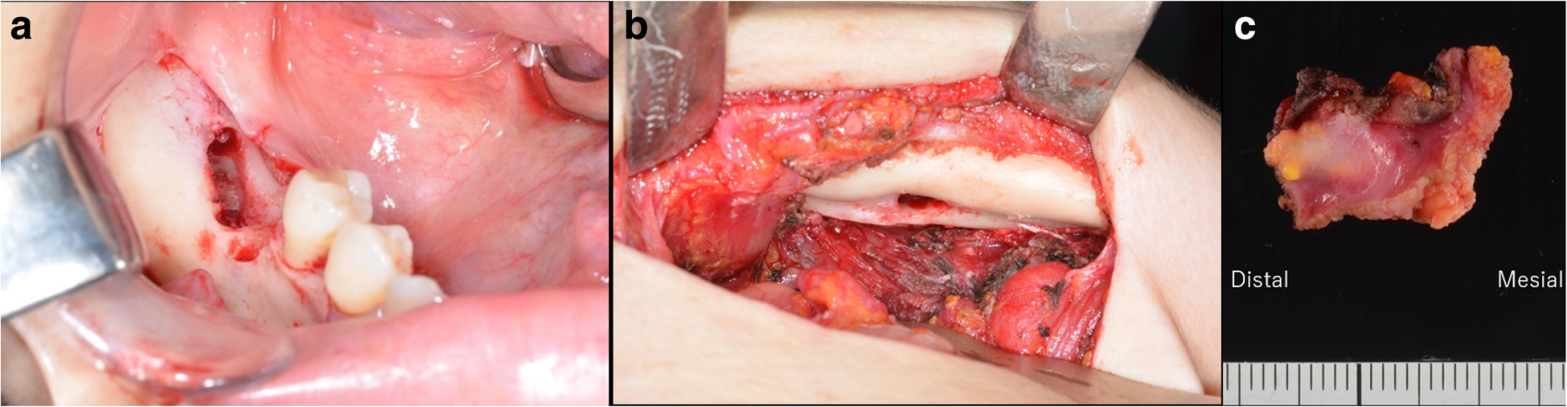
Perioperative images showing **a** the extraoral approach by submandibular incision and **b** the intraoral approach by gingival incision. **c** The surgical specimen

Surgical debridement under general anesthesia was performed. Via the submandibular region, an incision was made in the thickened periosteum at the inferior border of the mandible, after which the lingual periosteum was separated from the mandible (Fig. 3a). Tooth #46 was then extracted to achieve complete primary closure, and the gingiva incised (Fig. 3b) to enucleate the soft tissue including some of the hard tissues accompanying the periapical lesion of tooth #46 (Fig. 3c). There was no sequestrum and the intraoral wound was closed primarily. Our patient’s postoperative course was uneventful, with no exacerbation of the inflammation for more than 20 months.

**Fig. 4:**
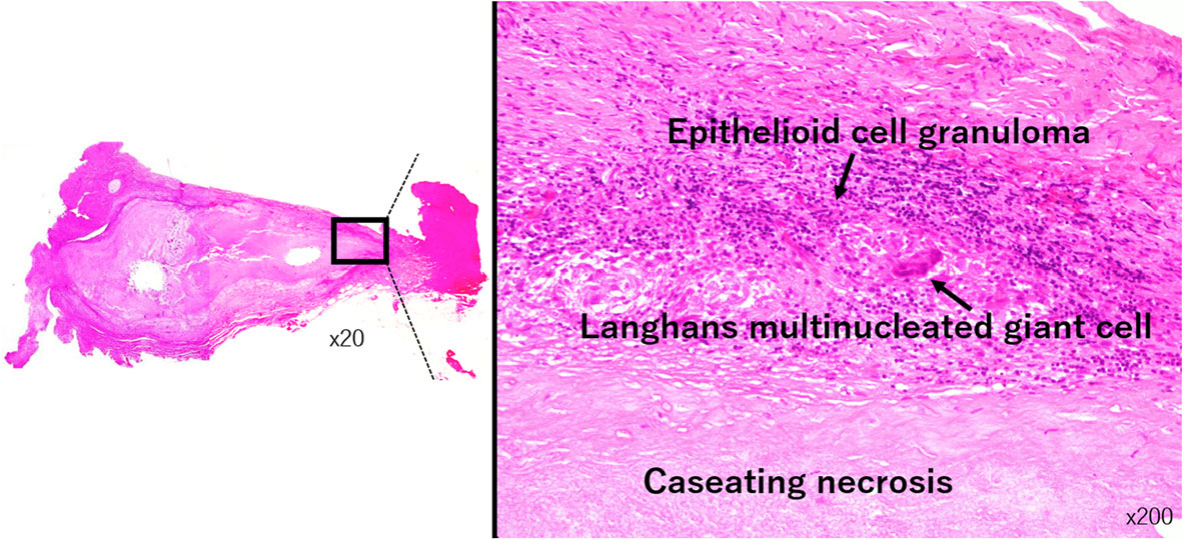
Hematoxylin-eosin staining revealed a central caseating necrosis surrounded by epithelioid cell granuloma in dense fibrous tissue

On histopathologic examination, a large epithelioid cell granuloma with central caseating necrosis was observed in the dense fibrous tissue (Fig. 4). The granuloma contained Langhans multinucleated giant cells. Acid-fast bacilli were not detected by either Ziehl–Neelsen staining or immunohistochemical staining using anti-BCG and anti-TB1 antibodies. Grocott staining revealed a slight presence of fungi. The pathological diagnosis was epithelioid cell granuloma including caseating necrosis.

## Discussion

Epithelioid cells are activated macrophages with a shape similar to that of epithelial cells. Epithelioid cell granuloma may occur at sites of intense inflammation and may be caseating or non-caseating. The caseating type occurs in the setting not only of specific inflammation, including tuberculosis and syphilis, but also of non-specific inflammation. The non-caseating type is seen in patients with sarcoidosis and leprosy, among other diseases.

Our patient did not present with the clinical findings typical of tuberculosis, that is, cough, sputum, and lymphadenopathy. She also had no abnormal findings on her chest X-ray and blood test. The pain had persisted for approximately 10 years, and there was no family history of tuberculosis. Acid-fast bacilli were not detected. Together, these findings ruled out tuberculosis. Syphilis was excluded by blood test.

There are very few reports in which a caseating epithelioid cell granuloma was induced by non-specific inflammation. Szilagyi *et al.* [8] reported the occurrence of a granuloma with caseation and Langhans-type giant cells in a tibia infected by *Salmonella typhi*, which was healed by saucerization. Assari *et al.* [9] described a chronic granulomatous reaction with caseating necrosis in the ulnar and radius of a patient with chronic recurrent multifocal osteomyelitis. The lesions were negative for bacteria, acid-fast bacilli, and fungi and were healed by non-steroid anti-inflammatory drugs and bisphosphonate.

In our patient with clinically diagnosed osteomyelitis of the mandible we chose treatment via surgical debridement. Osteomyelitis in the head and neck is induced by various factors. The incidence of osteomyelitis in the mandible and maxilla of patients with odontogenic predisposing factors is reported to be 22% and 30%, respectively [10]. Odontogenic osteomyelitis is usually induced by periodontitis. Chronic periapical periodontitis was shown to arise in association with an extraradicular bacterial biofilm located on an overextended gutta-percha surface or the cementum surface of the root against a background of periodontitis [3]. Planktonic bacteria emerging from biofilms may induce acute inflammation, but they are, for the most part, susceptible to antibiotics. However, bacteria encased in biofilms are resistant to antibiotics and to immune system attack and may therefore linger, even over the course of a lifetime [11]. *Porphyromonas gingivalis*, *Tannerella forsythensis*, and *Prevotella intermedia* are often detected in periapical biofilms [4], but many of the bacteria at these sites are, as yet, unidentified because they are uncultivative. While periapical periodontitis is mostly induced by infection of oral bacteria, fungi and viruses may also be involved. Fungi are isolated from the lesions of approximately 7% of patients with therapy-resistant periapical-periodontitis, with *Candida albicans* as the most commonly isolated species [5]. The presence of Epstein–Barr virus and human cytomegalovirus in chronic symptomatic periapical lesions has also been reported [6, 7]. Currently, the most effective treatment of bacterial biofilms and fungal and viral infections in a periapical lesion is surgical removal, although new therapies are being developed [11].

In our patient, periapical periodontitis might have induced the epithelioid cell granuloma with caseating necrosis, given the chronic dull pain that developed even after the root canal treatment of tooth #47. A dental X-ray before the extraction of tooth #47 showed insufficient root canal filling. We previously reported that Garrè’s osteomyelitis induced by periapical infection could be healed by a root canal procedure [12]. Had our patient presented with acute inflammation, she could have undergone retreatment, including curettage of the periapical granulation tissue with or without appendectomy or intentional replantation. Early treatment would have enabled the preservation of teeth #47 and #46 and avoided caseating granuloma formation. Acute inflammation might also have been controlled by our patient’s innate/acquired immune system, as she was young and healthy.

## Conclusions

This is the first report of a patient with neglected periapical periodontitis that may have induced an epithelioid cell granuloma with caseating necrosis in intra/extra mandibular bone. Treatment consisted of surgical enucleation but also required extraction of the vital adjacent tooth. In this patient, endodontic failure at the first stage made it impossible to control the inflammation locally, whether by retreatment, apicectomy, or even extraction, and surgical debridement under general anesthesia was therefore inevitable. This case well demonstrates the importance of appropriate and sufficient root canal treatments during the first stage of the procedure.

## References

[1] Chaudhary S, Kalra N, Gomber S. Tuberculous osteomyelitis of the mandible: a case report in a 4-year-old child. Oral Surg Oral Med Oral Pathol Oral Radiol Endod. 2004;97(5):603–6. 10.1016/S1079210404000241 15153873

[2] Signoretti FG, Endo MS, Gomes BP, Montagner F, Tosello FB, Jacinto RC. Persistent extraradicular infection in root-filled asymptomatic human tooth: scanning electron microscopic analysis and microbial investigation after apical microsurgery. J Endod. 2011;37(12):1696–700. 10.1016/j.joen.2011.09.018 22099908

[3] Noiri Y, Ehara A, Kawahara T, Takemura N, Ebisu S. Participation of bacterial biofilms in refractory and chronic periapical periodontitis. J Endod. 2002;28(10):679–83. 10.1097/00004770-200210000-00001 12398163

[4] Noguchi N, Noiri Y, Narimatsu M, Ebisu S. Identification and localization of extraradicular biofilm-forming bacteria associated with refractory endodontic pathogens. Appl Environ Microbiol. 2005;71(12):8738–43. 10.1128/AEM.71.12.8738-8743.2005 PMC1317348 16332869

[5] Waltimo TM, Sirén EK, Torkko HL, Olsen I, Haapasalo MP. Fungi in therapy-resistant apical periodontitis. Int Endod J. 1997;30(2):96–101. 10.1046/j.1365-2591.1997.00058.x 10332243

[6] Popovic J, Gasic J, Zivkovic S, Kesic L, Mitic A, Nikolic M, *et al.* Prevalence of Human Cytomegalovirus and Epstein-Barr Virus in Chronic Periapical Lesions. Intervirology. 2015;58(5):271–7. 10.1159/000441208 26551881

[7] Makino K, Takeichi O, Hatori K, Imai K, Ochiai K, Ogiso B. Epstein-Barr virus infection in chronically inflamed periapical granulomas. PLoS One. 2015;10(4):e0121548. 10.1371/journal.pone.0121548 PMC4401687 25884725

[8] Szilagyi A, Mendelson J, Portnoy J, Miller B. Caseating granulomas in chronic osteomyelitis: salmonellosis, tuberculosis or both? Can Med Assoc J. 1979;120(8):963–5. PMC1819244 436072

[9] Assari R, Ziaee V, Ahmadinejad Z, Vasei M, Moradinejad MH. Caseous granuloma: tuberculosis or chronic recurrent multifocal osteomyelitis? Iran J Pediatr. 2014;24(6):770–4. PMC4442841 26019785

[10] Prasad KC, Prasad SC, Mouli N, Agarwal S. Osteomyelitis in the head and neck. Acta Otolaryngol. 2007;127(2):194–205. 10.1080/00016480600818054 17364352

[11] Stewart PS, Costerton JW. Antibiotic resistance of bacteria in biofilms. Lancet. 2001;358(9276):135–8. 10.1016/s0140-6736(01)05321-1 11463434

[12] Ebihara A, Yoshioka T, Suda H. Garrè’s osteomyelitis managed by root canal treatment of a mandibular second molar: incorporation of computed tomography with 3D reconstruction in the diagnosis and monitoring of the disease. Int Endod J. 2005;38(4):255–61. 10.1111/j.1365-2591.2004.00920.x 15810976

